# Comparison of cognitive workload between very short answer questions and multiple-choice questions: an eye-tracking experiment

**DOI:** 10.1080/10872981.2026.2621434

**Published:** 2026-01-26

**Authors:** Maria Gabriela Carneiro Queiroz, Francisco Carlos Specian Junior, Pedro Tadao Hamamoto Filho, Thiago M. Santos, Stefan K. Schauber, Andrea M. Woltman, Dario Cecilio-Fernandes

**Affiliations:** a School of Medical Sciences, University of Campinas, Campinas, São Paulo, Brazil; b Departament of Neurosciences and Mental Health, Botucatu Medical School, Universidade Estadual Paulista (UNESP), Botucatu, São Paulo, Brazil; c Faculty of Medicine, Section for Health Professions Education (HELP) and Centre for Educational Measurement (CEMO/CREATE), Faculty of Educational Sciences, University of Oslo, Oslo, Norway; d Institute of Medical Education Research Rotterdam (iMERR), Erasmus MC University Medical Centre Rotterdam, Rotterdam, The Netherlands.

**Keywords:** Educational assessment, very short answer questions, eye tracking, medical education, cognitive workload

## Abstract

Very short answer questions (VSAQs) have gained attention for their superior psychometric properties compared to multiple-choice questions (MCQs). While VSAQs require knowledge recall, MCQs primarily involve knowledge recognition. This difference in cognitive processes may lead to varying cognitive workloads, defined as the amount of mental processing in working memory. Previous studies have not demonstrated consistent differences, likely due to reliance on self-reported measures. Eye tracking provides objective, process-level indicators of cognitive workload. This study investigated whether answering VSAQs requires a higher cognitive workload than answering MCQs. In a within-subject randomized crossover experiment, sixth-year medical students answered both VSAQs and MCQs. Cognitive workload was measured using screen-based eye tracking, focusing on the number of fixations and revisitations as objective indicators of mental effort. Data were analyzed using mixed-effects models. Thirty-four medical students participated, yielding 1,326 observations, which is the multiplication of the number of students by the number of questions (39 questions). Mixed-effects models showed a significant effect of question type on both workload indicators: VSAQs elicited more fixations and revisitations than MCQs (β_std = 0.30–0.39, *p* < .001). This effect remained after controlling for accuracy. Incorrect answers were associated with higher workload (β_std = −0.15–−0.16, *p* < .01). Heatmaps confirmed these findings, showing denser fixations on key diagnostic features for VSAQs and on answer options for MCQs. Answering VSAQs imposed a higher cognitive workload than MCQs. The presence of answer options in MCQs may reduce workload by providing unintentional cues, while VSAQs require active retrieval. Eye tracking proved valuable for distinguishing cognitive workload across assessment formats.

## Introduction

A fundamental choice in designing an educational assessment is selecting the appropriate question format for a test or exam. This choice can be informed by several factors, such as the format’s psychometric properties, its scalability and educational benefit. Recently, Very Short Answer Questions (VSAQs) have gained attention in medical education, since studies demonstrated that VSAQs possess clear advantages compared to multiple-choice questions (MCQs), including eliminating the possibilities for guessing the correct answer [1–5]. Although conceptual studies proposed that the response format is largely immaterial when questions are well-written and equivalent in number [6], several studies reported that VSAQs demonstrated higher reliability, discrimination and difficulty compared to MCQs [1–5]. Critically, with the advance and widespread accessibility of Large Language Models and Natural Language Processing, the burden of using human raters to score VSAQs answers will be an issue of the past. Thus, scoring VSAQs will become easily automated, decreasing the necessary effort and time for its correction [7]. However, deciding on the question format requires careful evaluation of the advantages and drawbacks in terms of evidence of the psychometric properties and educational benefit. Investigating whether VSAQs possess different educational benefit than MCQs could further strengthen the call to adopt VSAQs.

Findings from cognitive psychology research suggest that more effortful learning leads to better retention, than less effortful learning, known as desirable difficulties [8]. Although correctly answering both VSAQs and MCQs may require the necessary knowledge, the cognitive processing of retrieving the information may differ. The goal of VSAQs is to promote knowledge recall, whereas the widely used MCQs may rely on knowledge recognition. VSAQs require the knowledge that is related to the content to be available in the learners’ memory, whereas answering MCQs may rely on different strategies other than possessing the knowledge, from example, excluding other alternatives, recognising the correct alternatives and guessing. The difference between cognitive processing in answering both types of questions would reflect in the students’ cognitive workload while answering a question.

Cognitive workload is the amount of processing that occurs simultaneously in the students’ limited working memory [9,10]. A high cognitive workload indicates that students have difficulty in retrieving and processing all the necessary information when answering a question. For example, Specian et al. [11] demonstrated that complex questions require a higher cognitive workload than simple questions. The cognitive process of answering a VSAQs is thought to require a higher cognitive workload than MCQs, but research has demonstrated contradictory findings. Renes et al. did not find a significant difference in cognitive process between MCQs and a combination of different types of questions, including VSAQs. Schauber et al. [12] suggested that VSAQs and MCQs might involve different cognitive processes of clinical reasoning. Based on their findings, the authors hypothesised that MCQs might put higher demands on different cognitive skills, such as response inhibition and cognitive reflection, as compared to VSAQs [13]. A clear drawback of these studies was that they used traditional measures of response data such as self-reported confidence or response times.

An approach that offers clear benefits over traditional measures is eye-tracking, a method that captures eye movements while a person performs a task. Eye tracking has been used to objectively measure cognitive workload [14–17]. The recorded metrics of eye movements, along with pupil size and reactivity, can provide insights into cognitive processes that other methods cannot capture. The amount of cognitive workload has been often measured by the number of fixations and revisitations. Fixations occur when the eyes remain fixed for 100 to 500 milliseconds on a specific area, while revisitations refer to the number of times a person returns to a specific area. An increase in the number of fixations and revisitations is associated with an increase in cognitive workload [16,18–22]. As noted earlier, this is crucial since differences in cognitive workload are plausibly related to differences in the educational impact of different assessment formats.

Although answering a VSAQ is perceived to require a higher cognitive workload than MCQs [1,2,5,23], previous research using traditional methodologies have not shown a clear difference in cognitive workload between answering VSAQs and MCQs [12,13]. To our knowledge, no previous studies have investigated the difference in cognitive workload between answering VSAQs and MCQs using eye tracking. Therefore, our study sought to answer the following research question: Does answering VSAQs require a higher cognitive workload than answering MCQs? We hypothesised a higher cognitive workload in answering VSAQs compared to MCQs, indicated by a higher number of fixations and revisitations.

## Method

This is a prospectively within-subject randomised experiment with a crossover that compared the difference between cognitive workload in VSAQs and MCQs. In total, participants answered 40 questions with a maximum time of four minutes per question. Participants were randomised to start answering either VSAQs or MCQs to avoid the fatigue effect (MCQs → VSAQs or VSAQs → MCQs). Randomisation was conducted in a block of four students using an online randomisation tool. This randomisation method was selected to avoid discrepancies between groups during data collection. The participants were blinded to their group assignments and the nature of the other groups. Although the researcher who collected the data was aware of the randomisation, she had no influence or control over the intervention since it was conducted through pre-programmed software.

### Participants

We invited 59 sixth-year medical students from the University of Campinas to take part of this study, students who had experience with the format of both question types. The number of required participants in eye tracking can be determined by previous studies [24], which for knowledge assessment varies between 5 and 29 participants. Although there is no established minimum sample size for this type of study, our sample exceeds the size of most comparable investigations [11,24–26]. They were invited by the clinical teacher of the emergency medicine clerkship. Participation in this research was voluntary, and participants could withdraw from the research at any time. All participants signed the informed consent. This study was approved by the University's ethical committee (CAAE number: 71067423.3.0000.5404, reviewer number: 6.722.251), and all methods were performed following the relevant guidelines and regulations. The study was conducted in accordance with the principles stated in the Declaration of Helsinki.

### Data collection

The study was conducted in a controlled, isolated environment at the medical school to ensure no external stimuli interfered with the eye-tracking data. The experiment was performed on the same desktop computer (with 9th generation Intel i9 processor, dedicated 3050 video card, one terabyte HD, SSD for processing), equipped with dual high-resolution 19-inch screens. A control room adjacent to the testing environment allowed the researcher to monitor both the participants and the software. The experiment was conducted using Tobii Pro Nano 60 Hz, which captures eye movements during the test with an accuracy of 0.4 and spatial resolution of 0.10°. This device is a discrete object positioned at the bottom and front of the monitor. To calibrate the eye tracking, a nine-point calibration was used with 1 s each, following a path defined on the screen. The experiment only began once the calibration was completed and classified as either good or excellent according to the guidelines of the software. After the calibration, participants started the experiment in which they had to answer 40 questions, 20 VSAQs and 20 MCQs. Data collection was conducted individually. Although students were given up to two hours and 30 minutes to complete the task, the sessions lasted approximately one hour per participant.

### Testing material

**Figure 1.**
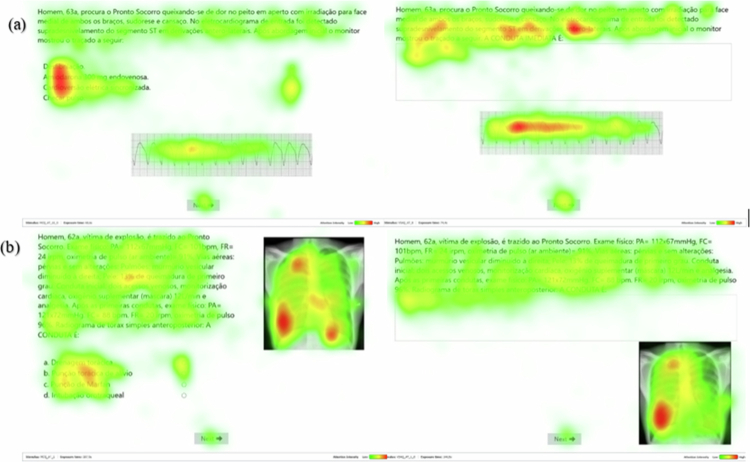
Eye-tracking heatmaps illustrating two clinical cases: (a) one case of ventricular tachycardia in which a MCQ was converted into a VSAQ, and (b) one case of pneumothorax in which a VSAQ was converted into a MCQ.

The experiment consisted of answering 40 questions retrieved from previous medical residency entry examinations. These items had been previously applied in the residency entry examination. The residency committee ensured the quality of the items by revising and selecting all items, and by conducting psychometric analyses. All selected items were deemed satisfactory after the residency entry examinations. We selected 20 VSAQs and 20 MCQs evenly distributed from two major areas: internal medicine and surgery. These domains were chosen because they are central to undergraduate medical education, are consistently covered in the core clinical curriculum, and closely align with the clinical training stage of the participating students. Including questions from both areas enhanced the representativeness of the assessment while avoiding over-reliance on a single content domain. As all participants had prior curricular and clinical exposure to both internal medicine and surgery, this selection minimised the potential influence of unequal familiarity on performance and supported the generalisability of the findings across core areas of medical training. To minimise the risk of systematic differences between formats, we transformed each VSAQ into a corresponding MCQ and vice versa, resulting in matched item pairs assessing the same content (example in Figure 1). Therefore, the comparison between type of questions was conducted within the same question content, with the only difference between the formats.

The selection and transformation of the questions were conducted by two co-authors, specialists in internal medicine (TMS) and surgery (PTHF). Both are experienced in knowledge assessment. The experts were responsible for creating the alternatives for VSAQs and adjusting questions to be as similar as possible in both formats. The questions were revised by a medical doctor who is also a master student and a psychometrician who has extensive experience with assessment in health professions education. Their review focused on evaluating content equivalence between formats, ensuring clarity and linguistic accuracy, and confirming that the transformed items adhered to established principles of test construction.

### Areas of interest (AOI)

The two specialists (TMS and PTHF) defined areas of interest in each of the questions for their specific field of expertise. These areas were marked based on key features - the essential information that is required to answer a question [27], such as findings from a clinical interview and/or a physical examination or the results of a diagnostic test [28]. We retrieved the number of fixations and revisitations within AOIs [11]. Non-relevant areas were excluded to avoid confounding the interpretation of eye-tracking data. Appendix 1 presents an illustrative example of the Areas of Interest (AOIs) defined for the analysis.

### Measures

#### Cognitive workload

Several eye-tracking metrics have been used to assess cognitive workload [16], but the number of fixations and revisitations are positively associated with increased cognitive workload [9,11]. We retrieved the number of fixations and revisitations on a specific area of interest (AOI). These measures reflect the extent of a test taker’s cognitive workload when answering questions and provide insight into their underlying cognitive processes while applying and integrating knowledge [9,16,29].

#### Accuracy

Participants’ responses to the VSAQs were transcribed into tables without revealing their experimental condition. Each answer was classified as either correct (1 point) or incorrect (0 points). Participants’ response to MCQs were automatically classified as correct (1 point) and incorrect (0 point), since there was only one correct alternative.

### Data analysis

For descriptive analysis we calculated means and correlations as appropriate. Also, we generated heatmaps to illustrate visual gaze patterns in two illustrative cases, allowing a qualitative comparison between the MCQ and VSAQ formats.

For investigating our research question, we analysed the data using mixed effects models. To account for the within-subject design of the current study, we included random effects for participants and cases to control for unexplained variance of the people and case difficulty. To compare the difference between VSAQs and MCQs on cognitive workload, we first estimated separate models to either fixations or revisitations as dependent variables and indicators of cognitive workload. We checked whether starting with different question format (MCQs → VSAQs or VSAQs → MCQs) led to overall differences between groups, and all models included the randomisation group as a control variable.

Across models, the independent variable was type of question (unconditional Model 1); we then added accuracy (Model 2) and its interaction with type-of-question (Model 3). Type of question, accuracy, and the randomisation group were entered as a factor. In total, six models were estimated—three for fixations and three for revisitations. To validate the findings using a single general model, we restructured the data to long format, so that both fixations and revisitations were included as an indicator of cognitive workload within the same model, while introducing a dummy variable for the type of indicator. To account for the different scales of the two indicators, we transformed them to T values, with a mean of M = 50 and a standard deviation of SD = 10. As a final check for the effect of the non-normality in the data on our findings, we performed an analysis using the log-transformed variables for the indicators of mental workload. Findings are reported in detail in the supplement (Table X; supplement) and notable differences are highlighted in the results section.

iMotions 10 software was used to collect, process, and store eye-tracking data. Data analysis was conducted using the R programming language for statistical computing (version 4.5.1). Linear mixed effects models were estimated using the lme4 package [30]. Correlations are described as small, medium, and large for a correlation coefficient of r = .10, r = .30, and r = .50, respectively [31].

## Results

Thirty-four sixth-year medical students agreed to participate in the study, which is 58% response rate. The participants' ages ranged from 24 to 59 years, with a mean of 26.73 years; 13 were female and 21 were male.

One of 40 questions was excluded because the eye tracking software did not collect the information for one of the groups. For the linear mixed models, our analysis included 1326 observations, which is given as the product of the number of participants (*N* = 34) by the number of questions (*N* = 39). The total number of correct answers for participant ranged from 12 to 29, with an average of 20.18.

### Descriptive statistics for cognitive workload

Descriptively, we observed on average M = 65 (SD 57) fixations in the MCQs condition as compared to M = 85 (SD 74) fixations for VSAQs. For revisitations, we found a similar pattern with M = 18 (SD 17) revisitations for MCQs and M = 27 (SD 26) for VSAQs. Revisitations and fixations were highly correlated (r = .84, *p* < 0.01). Mean accuracy was 47% correct for MCQs as compared to 51% correct for VSAQs. Across participants and conditions, there was a small, negative correlation between accuracy and fixation (r = −.12, *p* < 0.01) as well as between accuracy and revisitation (r = −.11, *p* < 0.01).

### Descriptive inspection of eye-tracking heatmaps

To give a better overview on the extra information gained from eye tracking, we chose two cases to illustrate the eye-tracking based measures, using heatmap. Since we did not formulate a priori hypotheses, we did not conduct test for statistical significance and treat these results as merely descriptive.

Figure 1a presents a heatmap comparing a MCQ and its corresponding VSAQ version. The English translations of the Figure 1 cases, including the AOIs delineated for each item, are provided in Appendix 1. Darker areas illustrate more fixations. Participants answering the VSAQ condition exhibited a higher density of fixations on key features of the case, such as “ST-segment elevation,” “anterolateral leads,” and on the ECG tracing itself. In comparison, for the same case in form of a MCQ, fixations tended to be concentrated on the answer options rather than on the clinical information within the case vignette. For the VSAQ participants responded correctly 43.8% of the time while for the MCQ condition, average accuracy was 62.5%. Furthermore, fixations were higher for the VSAQ (M_VSAQ_ = 74.7 vs. M_MCQ_ = 94.8) as were revisitations (M_VSAQ_ = 20.1 vs. M_MCQ_ = 30.7).

Figure 1b illustrates another clinical vignette showing a similar pattern to the previous case. This case was correctly solved by 18.8% of the participants in both the VSAQ and MCQ condition and thus appeared to be similarly difficult in both scenarios. Descriptively, however, the VSAQ-version of the case demanded a higher workload as suggested by higher number of fixations (M_VSAQ_ = 127.9 vs. M_MCQ_ = 75.9) and revisitations (M_VSAQ_ = 38.2 vs. M_MCQ_ = 19.3). As shown in the heatmap, when answering the MCQs version, participants’ fixations were dispersed across different regions of the chest radiograph, presumably influenced by the answer options, which included procedures such as *Marfan puncture*, *chest drainage*, and *orotracheal intubation*. This explains the higher fixation density near the cardiac apex. In contrast, in the VSAQ version, participants focused their gaze more consistently on the area corresponding to the right lung, where the pneumothorax was present, indicating more concise and targeted visual attention toward the key diagnostic feature.

In summary, these cases illustrate cases in which the cognitive workload for the VSAQ was, descriptively, higher than the cognitive workload for the MCQ. The first might suggest a pattern where participants “use” the response options to inspect the ECG, which might be a reason for this case being easier in the MCQ format. For the second case, there was a similar accuracy for both conditions, still the measures indicate the VSAQ to have a heavier cognitive workload than the MCQ version of the case.

### Does answering VSAQs require a higher cognitive workload than answering MCQs?

Mixed models indicated a significant effect of type of question on both indicators of cognitive workload with a standardised effect of β_std_ = 0.39 (CI 0.29–0.49; *p* < .001) for revisitations and β_std_ = 0.30 (CI 0.20–0.40; *p* < .001) for fixations, for the unconditional models (Models 1 and 4, Table 1). When we controlled for accuracy and its interaction with type of question (Models 3 and 6, Table 1), the estimate was β_std_ = 0.44 and β_std_ = 0.30 for revisitations and fixations, respectively. At the same time the results suggested a statistically significant effect for accuracy (β_std_ = −.17 and β_std_ = −.22 for revisitations and fixations; cf. Models 3 and 6, Table 1). Results show a consistent, moderate effect of Type of Question on cognitive workload. In addition, there is an effect of accuracy on workload indicating that incorrect questions were associated with higher cognitive workload. None of the models suggested a statistically significant effect for randomisation group were significant, indicating that randomisation was successful.

**Table 1.** Summary of seven mixed models fitted to the data, in increasing complexity.

Nr	Model formula	Type of Question (β_std_ [95% CI])	Accuracy (β_std_ [95% CI])	T-o-Q-Acc Interaction (β_std_ [95% CI])
1	Rev. ~ type-of-question	0.39 [0.29–0.49]		
2	Rev. ~ type-of-question + accuracy	0.38 [0.28–0.48]	−0.24 [−0.35–0.14]	
3	Rev ~ type-of-question + accuracy + t-o-c * accuracy	0.44 [0.32–0.56]	−0.17 [−0.31–0.04]	−0.14 [−0.34–0.04]
4	Fix. ~ type-of-question	0.30 [0.20–0.40]		
5	Fix. ~ type-of-question + accuracy	0.29 [0.19–0.39]	−0.24 [−0.33–0.15]	
6	Fix. ~ type-of-question + accuracy + t-o-c * accuracy	0.30 [0.19–0.41]	−0.22 [−0.35–0.10]	−0.03 [−0.20–0.14]
7	Single Model	0.37 [0.29–0.45]	−0.20 [−0.30–0.11]	−0.08 [−0.21–0.05]

Note: βstd is the standardised regression coefficient, comparable to a correlation coefficient. 95% CI is the 95% Confidence Interval, we interpret coefficients as statistically significant on the 5% level if do not include the zero. T-o-c is the type-of-question. The asterisk in the model formula indicates an interaction effect. The full model results for model 7 are attached as a supplement.

When we fitted a model that tested simultaneously for the effect of type of question and accuracy on cognitive workload (see supplementary material 1), we found a β_std_ = 0.37 (CI 0.29–0.44, *p* < .001) and the coefficient for accuracy was β_std_ = −0.20. When we estimated the same model using a logarithmic transformation of the dependent variable (i.e. cognitive workload), coefficients for both type of question and cognitive workload were largely comparable (β_std_ = 0.37 for the effect of type of question and β_std_ = −0.20 for accuracy).

**Figure 2.**
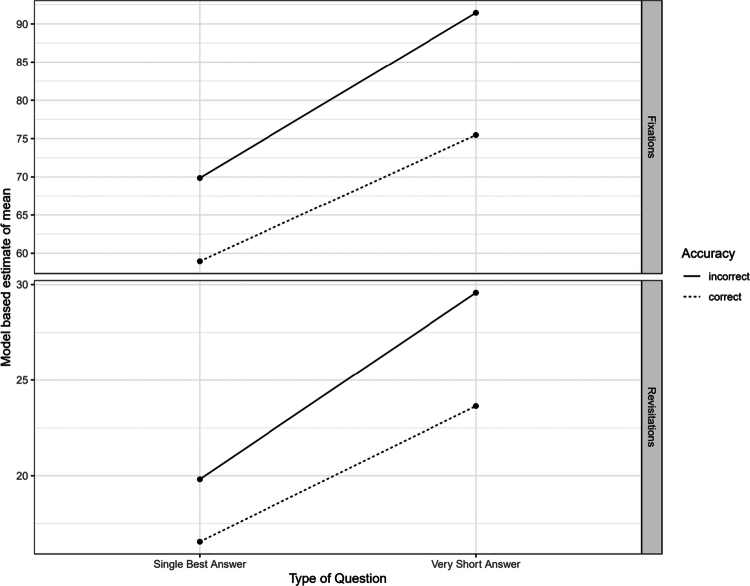
Model-based estimates for the effect of Type of Question on Mental Workload. For both indicators, MCQs in Single Best Answer Format show higher cognitive workload, and incorrect questions show higher workload across formats.

In summary, these findings indicate that VSAQ questions impose a higher cognitive workload than MCQs, even after accounting for possible differences in accuracy due to the response format. The according model-based estimates of the according means are given in Figure 2. Using Cohen’s classification [31], incorrect questions show a small effect of higher workload across both formats. For both revisitations and fixations, VSAQs format show higher cognitive workload, in the degree of a medium effect.

## Discussion

In this study, we compared the cognitive workload between VSAQs and MCQs. This is the first study to demonstrate that answering VSAQs requires a higher cognitive workload than MCQs, as measured by eye tracking. Our results differ from a previous study that found no difference in cognitive workload between different question types [12]. Our study has two important distinctions. First, our research design enables a direct comparison between VSAQs and MCQs. Second, we measured cognitive workload using eye tracking rather than relying on self-reported data.

Since the cognitive workload comparison was conducted within the same set of questions, our findings indicate that the alternatives in MCQs may unintentionally provide cues that reduce cognitive workload. Students may also use these alternatives to confirm their initial answers, an interpretation that we illustrated using heatmaps generated from participants’ gaze patterns in this study. In contrast, our findings are consistent with the view that answering a VSAQ requires students to retrieve information from their memory to verify their answer, which is associated with an increased cognitive workload. Again, this distinction is illustrated by the two examples of heatmaps we presented. In VSAQs, participants concentrated their gaze on the key features within the case, suggesting that they were actively retrieving and integrating relevant information to generate an answer. In contrast, during MCQs, participants tended to shift their attention between the case description and the answer options, indicating a more recognition-based and superficial search for matching cues rather than integrating the necessary knowledge to solve the question.

It is important to clarify, however, that our interpretation of cognitive workload should not be understood through the traditional tripartite framework of cognitive load theory (intrinsic, extraneous, and germane load). This distinction has been increasingly questioned, particularly because germane load is theoretically and empirically indistinguishable from intrinsic load, rendering the three-way categorisation conceptually problematic. More importantly, our study involved a performance task rather than a learning task: participants were not exposed to instructional materials, schema-construction activities, or design manipulations that would allow different types of cognitive load to be meaningfully separated [32,33]. Eye-tracking measures such as fixations and revisitations capture moment-to-moment cognitive processing within predefined areas of interest and do not reflect broader instructional influences or interface demands. Because AOIs were tightly controlled and restricted to the question stem, typing a brief answer did not introduce measurable visual search demands that would correspond to extraneous load. For these reasons, the differences observed between formats are best interpreted as reflecting differences in cognitive processing required to answer the items themselves, rather than distinct components of cognitive load.We found a higher cognitive workload for incorrect answers across all question types. Specian et al. also observed a higher cognitive workload for incorrect answers in MCQs. This higher cognitive workload indicates that students had to search their memory and that knowledge was not available. This aligns with the broader literature, which demonstrates that tasks requiring higher cognitive workload are associated with more errors.

Interestingly, mean accuracy was descriptively higher for VSAQs than for MCQs. While this difference was not statistically significant, it is still unusual given that previous research shows that VSAQs typically yield lower scores due to reduced cueing and guessing [5,34]. Our study differs from previous research in two ways, which might help explain this finding. Firstly, the questions were based on MCQs collected from a residency entry examination and were not specifically written for this study. That is, we transformed well-functioning MCQs into VSAQs by removing the distractors. Second, the test takers were in a phase of their training during which the study content matched their instruction. Hence, all testing content was directly relevant to their training. Taken together, several contextual factors might be related to response-format-dependent variation in item-level accuracy, rather than generic differences in difficulty between the formats. This would be, indeed, an interesting question for future research.Our findings may also have important implications for the educational benefit. Several studies demonstrated that students who are tested perform better on retention tests than those whose who simply re-study the learning material [35]. This is known as the testing effect [36,37], which requires students to actively retrieve information from their memory (recall). One of the most widely accepted explanations is the retrieval effort theory, which postulates that testing enhances retention because it requires cognitive effort to retrieve information. Also, the testing effect aligns with the concept of desirable difficulties, which holds that effortful learning leads to better retention than less effortful learning [37]. Since VSAQs require greater cognitive effort, we speculate that they could potentially enhance knowledge retention when used formatively during learning activities, aligned with retrieval practice. However, recent evidence suggests that this assumption must be treated cautiously. For example, both van Wijk et al. [5] and Lau et al. [34] found that VSAQs did not lead to superior knowledge retention when compared with MCQs, even in contexts specifically designed to promote retrieval practice. These findings indicate that increased cognitive effort alone may not be sufficient to generate measurable gains in retention. Further research is therefore needed to clarify under which conditions, and for which types of learners or content, VSAQs might confer learning advantages. This study has some limitations. First, we collected data from a single medical programme, focusing only on sixth-years medical students. Although cognitive workload is related to the development of expertise, we selected questions that were aligned with their expected level of knowledge. The questions were drawn from previous residency entry exams, which, in Brazil, are designed to assess students’ knowledge at the end of their undergraduate medical training. Comparisons to practice are also limited, as this study was performed in a controlled laboratory setting. For example, students had to answer a total of 40 questions, but most high-stakes examinations typically involve more than 80 questions. Finally, no systematic analysis of the heatmaps was performed; therefore, the visual analyses presented should be interpreted as illustrative rather than as quantitative evidence.

Our study has potential practical applications for assessment in health education. When selecting the question format, educators must align the assessment goals with factors such as format’s psychometric properties, scalability and educational benefit. Our findings provide educators new evidence that VSAQs require a higher cognitive workload than MCQs. Considering that VSAQs require a higher cognitive workload while increasing item’s difficulty and discrimination, high-stakes assessments should have a lower number of VSAQs compared to MCQs, otherwise the assessment may become too demanding for test-takers. Formative assessment should also prioritise VSAQs since we speculate, based on research in cognitive psychology, that the educational benefit may be greater than MCQs, but further research is necessary to further establish the impact of knowledge retention. MCQs should still be used by educators when a lower cognitive workload is desired, as an easy strategy to quickly identify gaps in learners` knowledge or as retrieval practice activity. Both VSAQs and MCQs can include, or omit, clinically relevant cues—and because either format can be transformed into the other while preserving the same underlying content—educators should focus on how items are written rather than on the format alone when aiming to align assessments with clinical reasoning. This suggests that improving item quality, ensuring clarity, and intentionally calibrating cue levels may be more impactful on assessment validity than simply choosing between VSAQs and MCQs. Although VSAQs demonstrate good reliability and discrimination, evidence regarding their ability to predict future clinical performance or long-term outcomes remains limited. This lack of predictive validity research represents an important gap in the literature and should be addressed in future studies. Finally, another practical implication is the use of eye tracking to investigate cognitive workload. Eye tracking systems are becoming more affordable and accessible [16], including with the use of a webcam [38]. This development will enable the investigation of cognitive workload in a more naturalistic setting.

## Conclusions

This study demonstrated that VSAQs require a higher cognitive workload compared to MCQs. Eye tracking proved to be a valuable tool for understanding and differentiating cognitive workload across different question types. These findings not only support the use of VSAQs in assessments but also highlight the potential of eye tracking to enhance our understanding of cognitive processes in test-taking, offering a powerful method for refining assessment designs.

## Supplementary Material

Supplement Model results.docx Supplement Model results.docx

## Data Availability

The datasets generated and analysed during the current study are not publicly available due to ethical and privacy restrictions related to participant confidentiality but are available from the corresponding author on reasonable request.

## Appendix 1- Translated Case Scenarios and Corresponding Areas of Interest (AOIs) for Figure 1

(a)

A 63-year-old man presents to the Emergency Department complaining of squeezing chest pain radiating to the medial aspect of both arms, accompanied by diaphoresis and fatigue. The initial electrocardiogram shows ST-segment elevation in the anterolateral leads. After the initial management, the cardiac monitor displays the following rhythm:

∘ Defibrillation
∘ Intravenous amiodarone 300 mg
∘ Synchronised cardioversion
∘ Check pulse

A 63-year-old man presents to the Emergency Department complaining of squeezing chest pain radiating to the medial aspect of both arms, accompanied by diaphoresis and fatigue. The initial electrocardiogram shows ST-segment elevation in the anterolateral leads. After the initial management, the cardiac monitor displays the following rhythm. THE IMMEDIATE MANAGEMENT IS:

(b)

A 62-year-old man, victim of an explosion, is brought to the Emergency Department. Physical examination: BP = 112/67 mmHg, HR = 101 bpm, RR = 24 breaths/min, pulse oximetry (room air) = 91%. Airway: patent and without abnormalities. Lungs: decreased breath sounds on the right side. Skin: 13% first-degree burns. Initial management includes two peripheral IV lines, cardiac monitoring, supplemental oxygen via face mask at 12 L/min, and analgesia. After initial stabilisation, physical examination shows: BP = 121/72 mmHg, HR = 88 bpm, RR = 20 breaths/min, pulse oximetry = 96%. A portable anteroposterior chest radiograph is obtained. THE APPROPRIATE MANAGEMENT IS:

a. Tube thoracostomy

b. Needle decompression

c. Marfan needle puncture

d. Orotracheal intubation

A 62-year-old man, victim of an explosion, is brought to the Emergency Department. Physical examination: BP = 112/67 mmHg, HR = 101 bpm, RR = 24 breaths/min, pulse oximetry (room air) = 91%. Airway: patent and without abnormalities. Lungs: decreased breath sounds on the right side. Skin: 13% first-degree burns. Initial management includes two peripheral IV lines, cardiac monitoring, supplemental oxygen via face mask at 12 L/min, and analgesia. After initial stabilisation, physical examination shows: BP = 121/72 mmHg, HR = 88 bpm, RR = 20 breaths/min, pulse oximetry = 96%. A portable anteroposterior chest radiograph is obtained. THE APPROPRIATE MANAGEMENT IS:

## References

[1] Mee J, Pandian R, Wolczynski J, et al. An experimental comparison of multiple-choice and short-answer questions on a high-stakes test for medical students. Adv in Health Sci Educ. 2024;29(3):783–801. doi: 10.1007/s10459-023-10266-3 PMC11208249 37665413

[2] Sam A, Field S, Collares C, et al. Very-short-answer questions: reliability, discrimination and acceptability. Med Educ. 2018;52(4):447–455. doi: 10.1111/medu.13504 29388317

[3] Sam A, Hameed S, Harris J, et al. Validity of very short answer versus single best answer questions for undergraduate assessment. BMC Med Educ. 2016;16:266. doi: 10.1186/s12909-016-0793-z 27737661 PMC5064885

[4] Sam A, Westacott R, Gurnell M, et al. Comparing single-best-answer and very-short-answer questions for the assessment of applied medical knowledge in 20 UK medical schools: cross-sectional study. BMJ Open. 2019;9(9):e032550. doi: 10.1136/bmjopen-2019-032550 PMC6773319 31558462

[5] van Wijk E, Janse R, Ruijter B, et al. Use of very short answer questions compared to multiple choice questions in undergraduate medical students: an external validation study. PLoS One. 2023;18(7):e0288558. doi: 10.1371/journal.pone.0288558 37450485 PMC10348524

[6] Schuwirth L, van der Vleuten C. Different written assessment methods: what can be said about their strengths and weaknesses? Med Educ. 2004;38(9):974–979. doi: 10.1111/j.1365-2929.2004.01916.x 15327679

[7] Clauser B, Yaneva V, Baldwin P, et al. Automated scoring of short-answer questions: a progress report. Appl. Meas. Educ. 2024;37(3):209–224. doi: 10.1080/08957347.2024.2386945

[8] Bjork R, Bjork E. Desirable difficulties in theory and practice. J Appl Res Mem Cogn. 2020;9(4):475–479. doi: 10.1016/j.jarmac.2020.09.003

[9] Borys M, Plechawska-Wójcik M, Wawrzyk M, et al. Classifying Cognitive Workload Using Eye Activity and EEG Features in Arithmetic Tasks. In: Damaševičius R, Mikašytė V, editors. Information and Software Technologies. ICIST 2017. Communications in Computer and Information Science. Springer, Cham. 2017. Vol. 756. 10.1007/978-3-319-67642-5_8

[10] Mayer R. Applying the science of learning to medical education. Med Educ. 2010;44(6):543–549. doi: 10.1111/j.1365-2923.2010.03624.x 20604850

[11] Specian Junior FC, Santos TM, Sandars J, et al. Identifying the response process validity of clinical vignette-type multiple choice questions: an eye-tracking study. Med Teach. 2023;45(8):845–851. doi: 10.1080/0142159X.2023.2182662 36840707

[12] Renes J, van der Vleuten C, Collares C. Utility of a multimodal computer-based assessment format for assessment with a higher degree of reliability and validity. Med Teach. 2023;45(4):433–441. doi: 10.1080/0142159X.2022.2137011 36306368

[13] Schauber S, Hautz S, Kämmer J, et al. Do different response formats affect how test takers approach a clinical reasoning task? An experimental study on antecedents of diagnostic accuracy using a constructed response and a selected response format. Adv Health Sci Educ. 2021;26(4):1339–1354. doi: 10.1007/s10459-021-10052-z PMC8452553 33977409

[14] Korbach A, Brünken R, Park B. Measurement of cognitive load in multimedia learning: a comparison of different objective measures. Instr Sci. 2017;45(4):515–536. doi: 10.1007/s11251-017-9413-5

[15] Korbach A, Brünken R, Park B. Differentiating different types of cognitive load: a comparison of different measures. Educ Psychol Rev. 2018;30(2):503–529. doi: 10.1007/s10648-017-9404-8

[16] Specian Junior FC, Litchfield D, Sandars J, et al. Use of eye tracking in medical education. Med Teach. 2024;46(11):1502–1509. doi: 10.1080/0142159X.2024.2316863 38382474

[17] Tien T, Pucher P, Sodergren M, et al. Eye tracking for skills assessment and training: a systematic review. J Surg Res. 2014;191(1):169–178. doi: 10.1016/j.jss.2014.04.032 24881471

[18] Dias R, Ngo-Howard M, Boskovski M, et al. Systematic review of measurement tools to assess surgeons' intraoperative cognitive workload. Br J Surg. 2018;105(5):491–501. doi: 10.1002/bjs.10795 29465749 PMC5878696

[19] Emhardt S, Kok E, van Gog T, et al. Visualizing a task performer's gaze to foster observers' performance and learning-a systematic literature review on eye movement modeling examples. Educ Psychol Rev. 2023;35(1):23. doi: 10.1007/s10648-023-09731-7

[20] Lai M, Tsai M, Yang F, et al. A review of using eye-tracking technology in exploring learning from 2000 to 2012. Educ Psychol Rev. 2013;10:90–115. doi: 10.1016/j.edurev.2013.10.001

[21] Van der Stigchel S, Hessels R, van Elst J, et al. The disengagement of visual attention in the gap paradigm across adolescence. Exp Brain Res. 2017;235(12):3585–3592. doi: 10.1007/s00221-017-5085-2 28884226 PMC5671527

[22] Wu W, Hall A, Braund H, et al. The development of visual expertise in ECG interpretation: an eye-tracking: augmented Re situ interview approach. Teach Learn Med. 2021;33(3):258–269. doi: 10.1080/10401334.2020.1844009 33302734

[23] Norman G, Swanson D, Case S. Conceptual and methodological issues in studies comparing assessment formats. Teach Learn Med. 1996;8(4):208–216. doi: 10.1080/10401339609539799

[24] Anderson B, Shyu CR. A preliminary study to understand tacit knowledge and visual routines of medical experts through gaze tracking. AMIA Annu Symp Proc. 2010;2010:21–25. 21346933 PMC3041324

[25] Yamada K, Augereau O, Kise K, et al. Estimation of confidence based on eye gaze: an application to multiple-choice questions In: Proceedings of the 2017 Acm International Joint Conference on Pervasive and Ubiquitous Computing and Proceedings of the 2017 Acm International Symposium on Wearable Computers (Ubicomp/Iswc '17 Adjunct). 2017. pp. 217–220. doi: 10.1145/3123024.3123138

[26] Yaneva V, Clauser B, Morales A, et al. Using eye-tracking data as part of the validity argument for multiple-choice questions: a demonstration. J Educ Meas. 2021;58(4):515–537. doi: 10.1111/jedm.12304

[27] Bordage G, Page G. The key-features approach to assess clinical decisions: validity evidence to date. Adv Health Sci Educ. 2018;23(5):1005–1036. doi: 10.1007/s10459-018-9830-5 29777464

[28] Lang VJ, Berman NB, Bronander K, et al. Validity evidence for a brief online key features examination in the internal medicine clerkship. Acad Med. 2019;94(2):259–266. doi: 10.1097/ACM.0000000000002506 30379661

[29] Goldberg J, Kotval X. Computer interface evaluation using eye movements: methods and constructs. Int J Ind Ergon. 1999;24:631–645. doi: 10.1016/S0169-8141(98)00068-7

[30] Bates D, Mächler M, Bolker B, et al. Fitting linear mixed-effects models using lme4. J Stat Softw. 2015;67(1):1–48. doi: 10.18637/jss.v067.i01

[31] Cohen J. A power primer. Psychol Bull. 1992;112(1):155–159. doi: 10.1037/0033-2909.112.1.155 19565683

[32] Kalyuga S. Cognitive load theory: how many types of load does it really need? Educ Psychol Rev. 2011;23(1):1–19. doi: 10.1007/s10648-010-9150-7

[33] Leppink J. Revisiting cognitive load theory: second thoughts and unaddressed questions. Scientia Medica. 2020;30(1):e-36918. doi: 10.15448/1980-6108.2020.1.36918

[34] Lau KY, Ang JYH, Rajalingam P. Very short answer questions in team-based learning: limited effect on peer elaboration and memory. Med Sci Educ. 2022;33(1):139–145. doi: 10.1007/s40670-022-01716-5 36569367 PMC9765373

[35] Yang C, Luo L, Vadillo M, et al. Testing (quizzing) boosts classroom learning: a systematic an meta-analytic review. Psychol Bull. 2021;147(4):399–435. doi: 10.1037/bul0000309 33683913

[36] Karpicke JD, Roediger HL. The critical importance of retrieval for learning. Sci. 2008;319(5865):966–968. doi: 10.1126/science.1152408 18276894

[37] Roediger HL, Karpicke JD. The power of testing memory basic research and implications for educational practice. Perspect Psychol Sci. 2006;1(3):181–210. doi: 10.1111/j.1745-6916.2006.00012.x 26151629

[38] Kaduk T, Goeke C, Finger H, et al. Webcam eye tracking close to laboratory standards: comparing a new webcam-based system and the EyeLink 1000. Behav Res Methods. 2023;56:5002–5022. doi: 10.3758/s13428-023-02237-8 37821751 PMC11289017

