# Supplementary material for: Comparison of cognitive workload between very short answer questions and multiple-choice questions: an eye-tracking experiment
Source: Med Educ Online. 2026 Jan 26;31(1):2621434. doi: 10.1080/10872981.2026.2621434 (PMC12849799; doi:10.1080/10872981.2026.2621434)
Supplement: Supplement Model results.docx [file ZMEO_A_2621434_SM8841.docx]

Supplementary Material 1. Model 7: Full model incorporating both indicators of cognitive workload simultaneously.

|  | **Model 7** | | | **Model 7, Interaction** | | |
| --- | --- | --- | --- | --- | --- | --- |
| *Predictors* | *std. β* | *standardized CI* | *p* | *std. β* | *standardized CI* | *p* |
| (Intercept) | -0.06 | -0.27 – 0.14 | 0.553 | -0.08 | -0.29 – 0.13 | 0.439 |
| Cognitive Workload, scaled [revisit] | -0.00 | -0.06 – 0.06 | 1.000 | 0.00 | -0.06 – 0.06 | 1.000 |
| Type-Of-Question [VSAQ] | 0.33 | 0.27 – 0.39 | **<0.001** | 0.37 | 0.29 – 0.45 | **<0.001** |
| Accuracy [Correct] | -0.24 | -0.32 – -0.17 | **<0.001** | -0.20 | -0.30 – -0.11 | **<0.001** |
| T-o-Q [VSAQ] × Accuracy [Correct] |  |  |  | -0.08 | -0.21 – 0.05 | 0.206 |
| **Random Effects** | | | | | | |
| σ^2^ | 0.61 | | | 0.61 | | |
| τ_00_ | 0.21 _question-id_ | | | 0.21 _question-id_ | | |
|  | 0.15 _respondent-id_ | | | 0.15 _respondent-id_ | | |
|  | 0.00 _randomizationblocks_ | | | 0.00 _randomizationblocks_ | | |
| N | 32 _respondent-id_ | | | 32 _respondent-id_ | | |
|  | 40 _question-id_ | | | 40 _question-id_ | | |
|  | 4 _randomizationblocks_ | | | 4 _randomizationblocks_ | | |
| Observations | 2550 | | | 2550 | | |
| Marginal R^2^ | 0.068 | | | 0.069 | | |
